# Bioorganic Chemistry: Current and Future Perspectives

**DOI:** 10.3390/molecules28165959

**Published:** 2023-08-09

**Authors:** Małgorzata Anna Marć, Enrique Domínguez-Álvarez, Claus Jacob

**Affiliations:** 1 Department of Technology and Biotechnology of Drugs, Jagiellonian University Medical College, Medyczna 9, 30-688 Kraków, Poland; 2 Instituto de Química Orgánica General (IQOG), CSIC, Juan de la Cierva 3, 28006 Madrid, Spain; 3 Division of Bioorganic Chemistry, School of Pharmacy, Saarland University, D-66123 Saarbruecken, Germany

Bioorganic Chemistry is an emerging field developing at the interface between the traditional fields of Chemistry and Biochemistry. As an interdisciplinary science, it also involves concepts from other fields, such as Physics, Biology, and Pharmacy. The field of Bioorganic Chemistry, to give a few examples (but not an exhaustive list), studies multiple different aspects, such as: the use of enzymes in synthesis; the synthesis of all types of biological molecules and biomolecules (sugars, lipids, proteins or peptides, nucleic acids, biopolymers, or their derivatives); the design and synthesis of ligands, inhibitors, or modulators of enzymes or any other cell biomolecules, cell membrane receptors, or targets; molecular recognition; biocatalysis; orthogonal synthesis; molecular and biological signaling; and informatics simulations of aspects related to cell molecules and biomolecules, both using molecular modelling and AI algorithms.

This Special Issue intends to gather perspective on the studies that are currently being developed in the field, in order to explore its recent developments. Researchers from many countries have contributed to this Special Issue with their work in the field of Bioorganic Chemistry: the different teams involved come from Asia (Vietnam), Europe (Austria, Germany, Italy, Poland, Russia, Slovak Republic, and Spain), North America (Canada and USA), and South America (Brazil). Together, they have contributed nine original articles and one review to this Special Issue.

The review published in this Special Issue, by Tien Anh et al. [1], explores the use of fluorination to design novel histone deacetylase inhibitors. This enzyme is essential to transcription: the deacetylation of histones enables a tighter binding to DNA, downregulating transcription. Finding more effective inhibitors of this enzyme, such as the ones reviewed, would enable the regulation of different genes involved in cancer; a strategy that has been successfully used to design inhibitors approved by the FDA for the treatment of specific types of cancer.

Regarding the experimental studies submitted to this Special Issue, multiple different approaches have been followed, leading to a very interdisciplinary Special Issue. Firstly, new methodologies have been developed to study the interactions between drugs and specific proteins, such as albumin [2], and to quantify the presence of double-stranded oligonucleotides [3]. Briefly, Aiello et al. [2] used nuclear magnetic resonance (NMR) spectroscopy to evaluate the interaction of anti-inflammatory drug salts (diclofenac sodium salt, ketorolac tris salt, and flurbiprofen sodium salt) with human serum albumin. Selective π pulses were applied to measure the relaxation rates, enabling the calculation of the affinity indexes for different protons of the drugs. The analysis of the results pointed out that diclofenac had the strongest binding, and that NMR can be used to study the drug-to-protein affinities, and the drug sites that participate in the interaction. In addition, Yuan, Dupuis, and Mekhssian [3] describe the successful application of a novel method to quantify double-stranded oligonucleotides as small interfering RNAs (siRNAs) in biological matrices by the use of a hybridized LC-MS/MS. This technique was previously used successfully to quantify single-stranded oligonucleotides, and this study extended this methodology to double-stranded ones. This novel methodology could be applied to both pharmacokinetic and toxicokinetic studies of siRNAs, and to the quantitative bioanalysis of these oligonucleotides.

A second group of contributions focuses on the cross-linking of DNA derivatives and proteins [4] and the synthesis of compounds with ability to bind with G-quadruplex structures [5]. In brief, Monakhova et al. [4] explored the covalent protein capture (cross-linking) of reactive DNA derivatives using the attachment of an acrylamide group to the sulfur of a cysteine amino acid and the binding of the C5 atom of 2′-deoxyuridine to an azide. The azide and the acrylamide are separated by a linker, and the study analyzed the influence of the linker length and structure on its cross-linking efficacy and selectivity. In another study, Mazzini et al. [5] reported the synthesis, characterization, and biological properties of novel kynurenic acid derivatives with a dihydroimidazoquinoline-3,5-dione core. It was determined that these compounds showed significant antiproliferative activity. Additionally, the mode of binding of the most cytostatic derivative to duplex and G-quadruplex structures was studied, determining that this compound showed a weak and non-specific interaction with duplex nucleotides, as well as a stable weak interaction with a G-quadruplex structure from human telomere.

The remaining contributions to this Special Issue on Bioorganic Chemistry focus either on the synthesis, characterization, and evaluation of novel bioactive synthetic compounds, or on the exploration of potential biological activities of previously reported synthetic compounds. This included studies that report potential advances in the search of compounds for photopharmacology [6], compounds with antioxidant activity [7], anticancer activity [8], and with applications in central nervous system diseases [9] or in vascular diseases [10]. Below, brief summaries of these studies are provided.

Borys et al. [6] designed and synthesized dibenzo[*b*,*f*]oxepine derivatives that were reacted with fluoroazobenzenes, and calculated *in silico* the energies of the HOMO and LUMO orbitals of the resulting compounds, and their binding to colchicine α and β-tubulin. The obtained results, combined with UV-Vis spectra, showed that these compounds could have potential applications in photopharmacology.

Kasungi Isika and Sadik [7] reported the synthesis and characterization of acetamide derivatives of natural antioxidants (quercetin, apigenin, and luteolin). These derivatives had, according to the computational tools used, an adequate drug-likeliness and a low toxicity score. Thus, the structural modification (i.e., addition of an acetamide) may improve the bioavailability of these compounds without reducing their antioxidant activity.

Oliveira et al. [8] synthesized and characterized 15 novel A-ring functionalized quinones containing two redox-active centers as anticancer and antioxidant agents. Many of them show promising IC_50_ values of below 500 nM in several cancer cell lines, thus validating this redox-active approach to designing active anticancer agents, enabling the preparation of novel potent anticancer derivatives.

Łażewska et al. [9] designed and synthesized a series of multitarget acetyl- and propionyl-phenoxy-pentyl(-hexyl) derivatives as acetylcholinesterase inhibitors, and as histamine H_3_ receptor (H_3_R) antagonists/inverse agonists. Two of the derivatives tested showed a very promising affinity for H_3_R in low nanomolar order, combined with a good ability to inhibit cholinesterase enzymes and a lack of toxic effects towards cells at concentrations below 50 µM.

Balis et al. [10] evaluated the ability of phthalic selenoanhydride to affect rat hemodynamic parameters and to determine its vasoactive properties in selected arteries. This compound showed a wide variety of biological effects in previous studies (e.g., anticancer, antibacterial, pro-apoptotic, antiviral, antibiofilm, and multidrug-resistance reversing activities) and potential additional applications are of interest. This compound transiently modulated most of the arterial pulse waveform parameters, whereas its sulfur and oxygen isosteres lacked this activity. Therefore, phthalic selenoanhydride can act on vascular smooth muscle cells.

These contributions provide a wide overview of the emerging interdisciplinary field of Bioorganic Chemistry, which is still under development. The Guests Editors of this Special Issue would like to thank the Authors and the Reviewers that have participated for gathering this varied collection of studies in the field.
